# Exosome-Bound WD Repeat Protein Monad Inhibits Breast Cancer Cell Invasion by Degrading Amphiregulin mRNA

**DOI:** 10.1371/journal.pone.0067326

**Published:** 2013-07-03

**Authors:** Makio Saeki, Hiroshi Egusa, Yuya Kamano, Yoshito Kakihara, Walid A. Houry, Hirofumi Yatani, Shinzaburo Noguchi, Yoshinori Kamisaki

**Affiliations:** 1 Department of Pharmacology, Graduate School of Dentistry, Osaka University, Suita, Osaka, Japan; 2 Department of Fixed Prosthodontics, Graduate School of Dentistry, Osaka University, Suita, Osaka, Japan; 3 Department of Biochemistry, University of Toronto, Toronto, Ontario, Canada; 4 Department of Breast and Endocrine Surgery, Graduate School of Medicine, Osaka University, Suita, Osaka, Japan; Wayne State University, United States of America

## Abstract

Increased stabilization of mRNA coding for key cancer genes can contribute to invasiveness. This is achieved by down-regulation of exosome cofactors, which bind to 3'-UTR in cancer-related genes. Here, we identified amphiregulin, an EGFR ligand, as a target of WD repeat protein Monad, a component of R2TP/prefoldin-like complex, in MDA-MB-231 breast cancer cells. Monad specifically interacted with both the 3'-UTR of amphiregulin mRNA and the RNA degrading exosome, and enhanced decay of amphiregulin transcripts. Knockdown of Monad increased invasion and this effect was abolished with anti-amphiregulin neutralizing antibody. These results suggest that Monad could prevent amphiregulin-mediated invasion by degrading amphiregulin mRNA.

## Introduction

Invasiveness of cancer cells is one of the hallmarks of malignant progression evidenced by local invasion and distant metastasis [1]. Despite its central role in cancer progression, the molecular mechanism driving tumor cell invasion is not totally understood. The epidermal growth factor receptor (EGFR) is a transmembrane protein possessing intrinsic tyrosine kinase activity. There are several EGF family ligands that can bind and activate the EGFR, including EGF and amphiregulin. Accumulated evidence suggests that EGFR and its ligands are involved not only in cell proliferation but also in metastatic phenotype such as cell invasion. In breast cancers, the EGFR protein is overexpressed and is considered a potential therapeutic target [2], [3]. Amphiregulin overexpression has also been demonstrated in breast cancer [4]. However, the genetic alterations responsible for this overexpression remain unknown.

The R2TP complex, identified in yeast by systematic proteomic and genomic approaches, is a multi-subunit Hsp90 interacting complex formed by Rvb1, Rvb2, Tah1, and Pih1 [5], [6]. Rvb1 and Rvb2 (Pontin and Reptin in human) are evolutionarily highly conserved AAA+ ATPases and have been reported to be overexpressed in several cancers including hepatocellular carcinoma, colon and bladder cancer, and melanoma; they are potential targets for cancer therapy [7]. WD repeat protein 92 (WDR92), also known as Monad [8], has been reported to be a component of chaperone-related prefoldin complex, called R2TP/prefoldin-like complex which is one of the counterparts of simple yeast R2TP complex [9]. Previously, we co-purified four Monad binding proteins; RNA polymerase II-associated protein 3 (RPAP3, Tah1 in yeast), Pontin, Reptin, and PIH1 domain containing protein 1 (PIH1D1, Pih1 in yeast), [10]–[14], indicating that Monad interacts with the core R2TP complex. We have also reported that Monad enhances apoptosis induced by tumor necrosis factor [8], raising the possibility that Monad has tumor-suppressive function. However, the role of Monad in cancer has not been clarified.

WD repeats are homologous sequences of 40 amino acids frequently bracketed by the amino acid pairs Gly-His on the amino end of the repeat and Trp-Asp at the carboxyl end and are almost exclusive to eukaryotes [15]. They are generally thought to play a role in protein-protein interactions. Recently, Lau et al. identified WD repeat as a previously undescribed RNA binding domain and suggested that WD repeat should be considered as predictive of potential function in RNA binding [16].

Although relatively under-studied compared to transcriptional regulation, it is becoming increasingly clear that mRNA stability is an important control point in the regulation of gene expression [17]. Understanding the mechanisms that regulate mRNA turnover requires the identification of the enzymatic machinery for mRNA degradation. The exosome is a multi-protein complex displaying RNA degradation activity and plays an important role in the coordination of diverse processes in RNA metabolism [18]. In the nucleus, the exosome plays a crucial role in the proper maturation of RNA such as ribosomal RNA (rRNA) and small nucleolar RNA (snoRNA). The Hsp90 and R2TP complex have been found to be required for pre-rRNA processing and snoRNA accumulation [6].

In the cytoplasm, the exosome takes part in general mRNA turnover and in more specialized pathways of mRNA decay, such as the regulated degradation of transcripts and RNA surveillance pathways, preventing translation of aberrant mRNA molecules. The best characterized of these mRNA quality control systems is the accelerated degradation of transcripts containing premature termination codons (PTCs), known as nonsense-mediated decay (NMD). SMG-1 is a component of the mRNA surveillance complex involved in NMD and, hence, regulates the degradation of mRNAs containing PTCs. A function of the R2TP complex components, Pontin and Reptin, in the regulation of SMG-1 activity has been recently reported [19].

mRNA stability is governed by orchestrated interactions between sequence and/or structural elements (*cis* elements) in mRNAs and specific *trans*-acting factors that recognize these elements. The best-characterized *cis*-acting sequences responsible for mRNA decay in mammalian cells are the AU-rich elements (ARE) present in the 3′-untranslated region (3′-UTR) of mRNA (20). ARE-regulated mRNA decay in mammalian cells appears to occur predominantly via exosome-mediated 3′-to-5′ degradation [21], [22]. During the preparation of the manuscript, it has been reported that amphiregulin mRNA is stabilized by ARE-binding protein, Human antigen R (HuR, [23]).

In the present study, we found that in human breast cancer cells, Monad binds to one of the exosome component, OIP2, raising the possibility that Monad may recruit the exosome for mRNA turnover. We also found that Monad binds to and regulates amphiregulin mRNA stability. Thus, we provide evidence that Monad prevents breast cancer cell invasion by degrading amphiregulin mRNA.

## Materials and Methods

### Materials

Anti-OIP2 antibody was from Proteintech. Anti-GAPDH (glyceraldehyde-3-phosphate dehydrogenase, 23040091) antibody was from Chemicon. Anti-Monad antibody was previously described [8]. Actinomycin D was from Calbiochem.

### Cell Culture and Knockdown Experiments

MDA-MB-231 cells or MCF-7 cells were cultured in DMEM supplemented with 10% heat-inactivated fetal bovine serum containing 100 µg/ml streptomycin, 100 IU/ml penicillin, and 1 µl/ml amphotericin B. Cell proliferation was measured by MTT (Sigma, M2128) assay. Monad, RPAP3 or PIH1D1-specific siRNA was purchased from Qiagen and targeted the following sequences: 5′-ACGGTGGGAGACAAACATCAA-3′, 5′-TTGAAGGATAGTTCTGTCGAA-3′ or 5′-CAGATGCTAGAGGAGGACCAA-3′, and transfected with Lipofectamine RNAiMAX according to the manufacturer’s instructions (Invitrogen). AllStars Negative Control siRNA (Qiagen) was used as a control.

### Lentiviral Transduction and Establishment of Stable Cell Line

The PCR-amplified coding sequence for human or mouse Monad was cloned into pENTR/D TOPO and subcloned into pLenti6.3//V5-DEST or pLenti6.3/TO/V5-DEST using Gateway System (Invitrogen). These vectors or pLenti3.3/TR were cotransfected into 293FT cells with ViraPower packaging mix (Invitrogen) to generate the lentivirus according to manufacturer’s protocol. Cells were transduced with the lentivirus and stable cell lines were generated by selecting with blasticidin.

### Immunoblotting and Immunoprecipitation

Cells were lysed in extraction buffer (1% Triton X-100, 120 mM NaCl, 5 mM EDTA, 10% glycerol and 20 mM Tris, pH 7.4) including protease inhibitor cocktail (Roche). Total protein was mixed with Laemmli buffer, separated by SDS-PAGE and transferred to PVDF membranes (Millipore Corporation). Immunoblotting and immunoprecipitaion were carried out as described previously [24], [25]. Briefly, equal protein concentrations of lysates were incubated with antibody for 16 h, followed by incubation with Protein G Sepharose (Amersham Biosciences) for 1 h. The Sepharose beads were washed five times with the buffer described above, associated proteins were recovered by boiling in Laemmli buffer.

### Enzyme-linked Immunosorbent Assay (ELISA)

Conditioned medium was obtained from MDA-MB-231 cells grown in six-well plates. An AREG DuoSet ELISA (R&D Systems) was used to measure amphiregulin medium concentration following the manufacturer's instructions.

### DNA Microarray

Total RNA samples were isolated from MDA-MB-231cells using TRIzol (Invitrogen). Each RNA sample was labeled with Cy3 and then hybridized with CodeLink Human Whole Genome Bioarray (Applied microarrays), and the signals were detected with GenePix4000B (Olympus). Microarray Data Analysis Tool version 3 software (Filgen) was used to normalize and analyze the expression data of each gene.

### RNA Isolation, cDNA Synthesis, and Real-time PCR

Total RNA was extracted using TRIzol (Invitrogen), and reverse-transcribed with SuperScript First-Strand Synthesis System (Invitrogen) according to the manufacturer’s protocol. TaqMan Gene Expression Assay based real-time PCR was performed with an ABI PRISM 7900 sequence detection system (Applied Biosystems). Each TaqMan assay was conducted in four replicates for each RNA sample. They were assayed with Universal PCR Master Mix using universal cycling conditions (10 min at 95°C; 15 s at 95°C, 1 min at 60°C, 40 cycles). The TaqMan probe/primer sets for the endogenous control and target genes were as follows: GAPDH, Hs99999905_m1; RPAP3, Hs00226298_m1; PIH1D1, Hs00215579_m1; Monad, Hs00399034_m1; amphiregulin, Hs00950669_m1; MMP1, Hs00899658_m1; uPA, Hs01547054_m1. Results are expressed as relative abundance of mRNA normalized to an internal control (GAPDH).

### 5-Ethnyluridine (EU) Pulse-labeling

Analysis of RNA half-life was performed by EU pulse-labeling of RNA using the Click-iT Nascent RNA Capture Kit (Invitrogen). EU was added to medium and incubated for 24 h. At indicated time points after replacing EU-containing medium with EU-free medium, cells were harvested. EU-labeled RNAs were biotinylated and captured using the Click-iT Nascent RNA Capture Kit (Invitrogen), according to the manufacturer's instructions. Isolated RNAs were used for real-time RT-PCR.

### Immunoprecipitation of Monad-associated mRNAs

MDA-MB-231 cells were lysed with polysome lysis buffer (100 mM KCl, 5 mM MgCl_2_, 10 mM HEPES, pH 7.0, 0.5% Nonidet P-40) containing RNase inhibitors and EDTA-free proteinase inhibitors. A total of 100 µl of lysate was incubated for 2 h with A/G agarose beads (Pierce) precoated with 20 µg of anti-Monad or normal rabbit IgG (Santa Cruz). After washing, the beads were incubated in the buffer containing 0.1% SDS and 0.5 mg/ml proteinase K (15 min, 55 °C) to digest the protein bound to the beads. RNA was subject to real-time RT-PCR.

### Promoter and 3′-UTR Luciferase Assay

Luciferase reporter construct containing the promoter or 3′-UTR of amphiregulin (pLightSwith vector, Switchgear Genomics) was transfected in MDA-MB-231 cells. Cell extracts were prepared 24 h after transfection, and luciferase activity was measured using the Luciferase Reporter Assay System (Promega).

### In vitro Transcription and RNA Pull-down Assay

Sense and antisense strand of 3′-UTR of amphiregulin (Switchgear Genomics) was subcloned in pGEM-T Easy (Promega) downstream of the T7 promoter. Linearized pGEM-T vector was in vitro transcribed with T7 polymerase in the presence of 5-Bromo-UTP (BrU, MBL). For RNA pull-down assay, BrU-labeled RNA was immunoaffinity-purified with anti-BrdU antibody, which cross-reacts with BrU using RiboTrap Kit (MBL) according to the manufacturer’s protocol. Recombinant Monad was described previously [8].

### Cell Invasion Assay

Invasive potential of MDA-MB-231 cells was assayed by a Boyden chamber method. Suspensions of cells in DMEM were added to the upper well of BioCoat Matrigel chambers (BD Biosciences) containing an 8 µM porous membrane, and incubated for 24 h. Cells that had degraded the Matrigel and passed through the porous membrane were fixed with methanol, stained with haematoxylin and eosin and counted under a light microscope in five random fields/membrane. Each assay was done in triplicate. For the ligand blocking studies, anti-human AREG antibody (MAB262, R&D Systems) was resuspended in PBS and applied to cells at a concentration of 1 µg/ml.

### Statistical Analysis

Data are expressed as mean ± S.E. Statistical differences between groups were determined using Tukey test after ANOVA. P<0.05 was considered significant.

## Results

### Identification of Monad Downstream Target Gene by Comprehensive Screen of R2TP Complex Target Molecules

We previously identified the proteins of the R2TP complex as major binding partners of Monad [10]. Therefore, we hypothesized that the effect of Monad on breast cancer cells could be R2TP complex-dependent. We have recently reported that two core components of the R2TP complex, RPAP3 and PIH1D1 stabilize post-translationally each other [12]. From these findings, we expected that the depletion of RPAP3 or PIH1D1 would result in the similar cellular phenotype. To test this hypothesis, we attempted to identify possible Monad-R2TP complex target genes in MDA-MB-231 cells which were treated with either RPAP3 or PIH1D1 siRNA by using microarray analysis. The lists of top ten genes up-regulated in RPAP3 or PIH1D1 siRNA-treated cells are given in Tables 1 and 2. Of these, we focused on amphiregulin, because its probe signal was markedly up-regulated both in RPAP3 and PIH1D1 siRNA-treated cells; moreover, amphiregulin has been reported to have high levels of expression in hormone therapy-resistant breast cancer cells [4], [26], [27].

**Table 1:** Up-regulated gene in RPAP3-knockdown MDA-MB-231 cells.

Gene Symbol	Gene Name	GeneBank Accession	Fold increase
MMP1	matrix metalloprotease 1	NM_002421	15.6
AREG	amphiregulin	NM_001657	4.5
TM4SF1	transmembrane 4L	NM_014220	3.3
SMAGP	small cell adhesion glycoprotein	NM_001031628	3.3
CTSC	cathepsin C	NM_001114173	3.1
CTSB	cathepsin B	NM_001908	3.0
ITGA6	integrin, alpha 6	NM_000210	2.9
RPS10	ribosomal protein S10	NM_001014	2.9
C7orf10	chromosome 7 open reading frame 10	NM_024728	2.8
GSTO1	glutathione S-transferase omega 1	NM_004832	2.7

**Table 2:** Up-regulated gene in PIH1D1-knockdown MDA-MB-231 cells.

Gene Symbol	Gene Name	GeneBank Accession	Fold increase
PLAU	plasminogen activator, urokinase	NM_001145031	3.0
SPANXC	SPANX family, member C	NM_022661	3.0
HISTIH2AA3	histone cluster2, H2aa3	NM_003516	2.7
AREG	amphiregulin	NM_001657	2.5
SFTA1P	surfactant associated 1 (pseudogene)	NR_027082	2.2
HIST1H2BC	histone cluster 1, H2bc	NM_003526	2.2
HIST1H2BJ	histone cluster 1, H2bj	NM_021058	2.2
HIST1H4H	histone cluster 1, H4h	NM_003543	2.1
HIST1H2BD	histone cluster 1, H2bd	NM_021063	2.1
NEFL	neurofilament, light polypeptide	NM_006158	2.1

**Figure 1:**
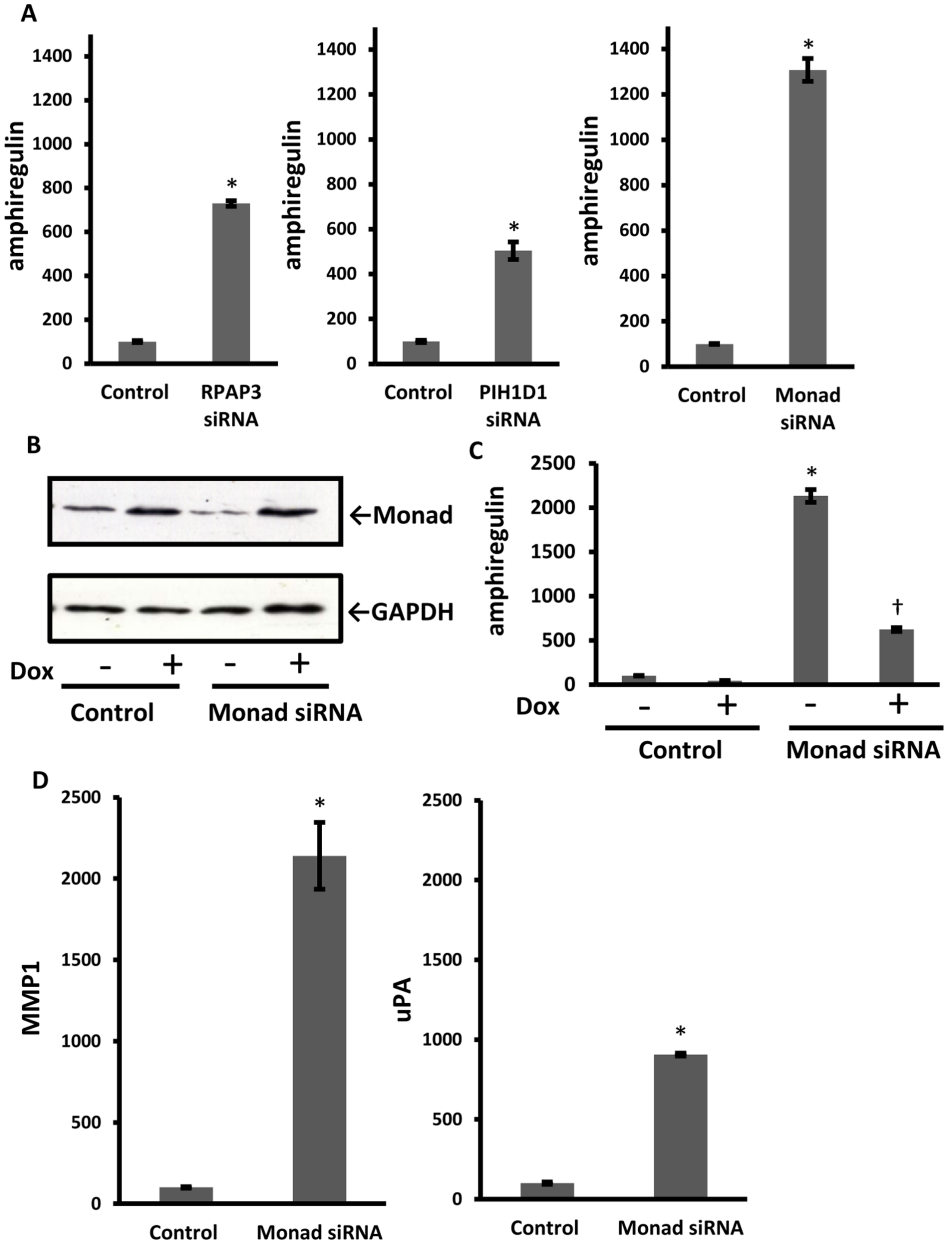
Knockdown of Monad increased amphiregulin mRNA. (A, C, D) Relative mRNA levels were analyzed by real-time RT-PCR at 48 h after the treatment of MDA-MB-231 cells with the indicated siRNA and expressed as percentage to that of control siRNA-treated cells from four independent experiments (mean ± S.E.). Data were normalized based on GAPDH mRNA copy numbers. (B, C) Mouse Monad was overexpressed by doxycycline (Dox). Monad protein levels (B) and relative amphiregulin mRNA levels (C) were analyzed by immunoblotting and real-time RT-PCR, respectively, at 48 h after the treatment of Dox-regulated MDA-MB-231 cells with control or Monad siRNA. *P<0.01 vs. control. ^†^ *P*<0.01 vs. Dox (−).

To confirm the results from microarray analysis, amphiregulin expression was examined by real-time RT-PCR. In concert with the microarray expression profiles, treatment of MDA-MB-231 cells with either RPAP3 or PIH1D1 siRNA significantly up-regulated the amphiregulin expression (Fig. 1A). As expected, treatment with Monad siRNA also significantly up-regulated the amphiregulin expression (Fig. 1A). To rule out off-target effects of siRNA, we used a rescue strategy. We established doxycycline (Dox)-regulatable mouse Monad-overexpressing MDA-MB-231 cells and made the induced mRNA insensitive to Monad siRNA (Fig. 1B). As shown in Fig. 1C, induced overexpression of Monad by Dox decreased amphiregulin expression (Dox +, Control), and Monad knockdown effect was partially rescued in Monad-expressing cells (Dox +, Monad siRNA). We also confirmed that matrix metalloprotease (MMP) 1 and urokinase-type plasminogen activator (uPA), up-regulated genes in the RPAP3 or PIH1D1 knockdown microarray, respectively, were indeed increased by Monad knockdown (Fig. 1D).

### Monad Regulates the Amphiregulin Secretion in Human Breast Cancer Cells

**Figure 2:**
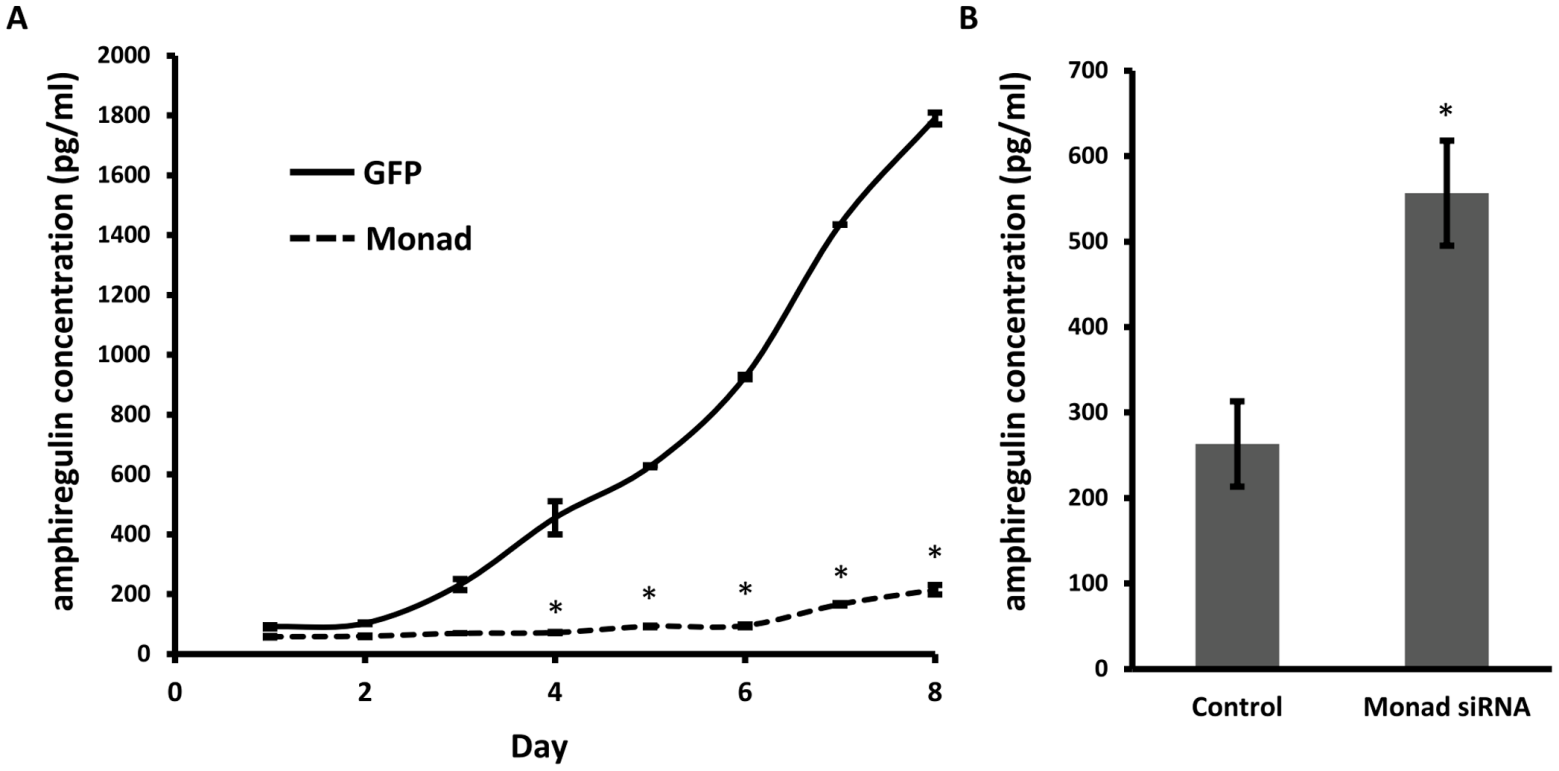
Secretion of amphiregulin is regulated by Monad. Amphiregulin secreted from MDA-MB-231cells overexpressing GFP or Monad (A) and treated with control or Monad siRNA (B) was measured by an ELISA. The amphiregulin concentration in the medium was normalized to cell number. Error bars represent the S.E. of three independent experiments. *P<0.01 vs. control.

The biologically active amphiregulin protein is released from the precursor transmembrane amphiregulin by proteolytic activation [4]. To detect secreted amphiregulin, we used an ELISA. The conditional media of MDA-MB-231 cells overexpressing GFP (control) or Monad were analyzed in 24-h intervals. The level of amphiregulin was lower in Monad-overexprssing cells relative to control cells (Fig. 2A). Conversely, after 48-h treatment of Monad siRNA, amphiregulin secretion was increased (Fig. 2B).

### Knockdown of Monad Stabilizes Amphiregulin mRNA

**Figure 3:**
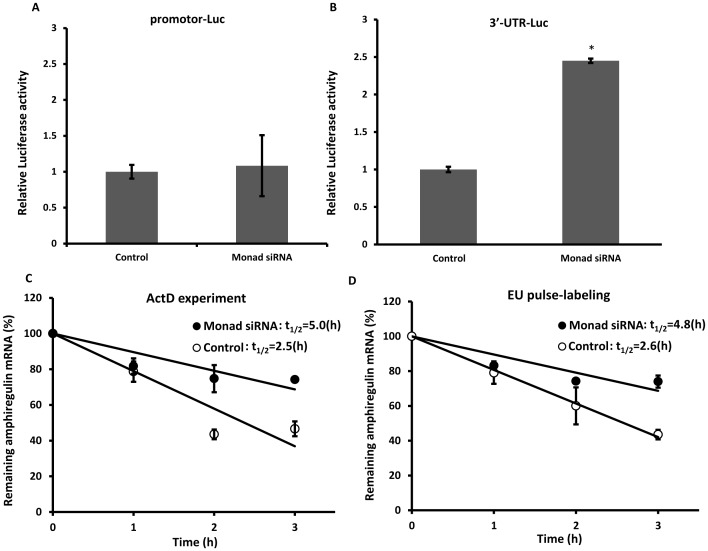
Monad knockdown stabilizes amphiregulin mRNA. MDA-MB-231 cells were transfected with amphiregulin promoter (A) or 3′-UTR (B) -luciferase (Luc) reporters and after 2 days they were analyzed using luciferase reporter assays. The results represent the mean values with S.E. from three independent experiments. (C) MDA-MB-231cells were treated with either control or Monad siRNA and after 2 days with 5 µg/ml of actinomycin D (ActD). Relative amphiregulin mRNA levels at the different time points were analyzed by real-time RT-PCR and expressed as percentages of the level at the 0-h time point from four independent experiments (mean ± S.E.). Data were normalized based on GAPDH mRNA copy numbers. (D) The decay rates of amphiregulin were determined using the Click-iT Nascent RNA Capture Kit (Invitrogen) and expressed as percentages of the level at the 0-h time point from four independent experiments (mean ± S.E.). Data were normalized based on GAPDH mRNA copy numbers.

Increased mRNA accumulation is due to either increased transcription or increased RNA stability. Since transcriptional regulation of amphiregulin has been reported [28], [29], we first investigated whether knockdown of Monad increases amphiregulin promoter activity. Transient expression assays, using a promoter-reporter construct, showed very little changes in amphiregulin promoter activity after Monad siRNA treatment (Fig. 3A), suggesting that the increased amphiregulin accumulation may be due to the stabilization of amphiregulin mRNA rather than augmentation of amphiregulin transcription. Consistent with this hypothesis, the activity of the luciferase which has amphiregulin 3′-UTR was much higher in Monad knockdown cells as measured by the luciferase-reporter assay (Fig. 3B). Actinomycin D time course experiments were also performed to determine the decay rate of amphiregulin mRNA. As shown in Fig. 3C, knockdown of Monad prolonged the half-life of amphiregulin mRNA to two times longer than the control (5.0 h versus 2.5 h). We also used inhibitor-free method, EU pulse-labeling, which is commercially available as the Click-iT Nascent RNA Capture Kit (Invitrogen) and confirmed that amphiregulin mRNA was stabilized by siRNA treatment of Monad (4.8 h versus 2.6 h, Fig. 3D). Altogether these results indicate that Monad controls amphiregulin mRNA levels, at least in part, through regulation of its mRNA stability.

### Binding of Monad to Amphiregulin mRNA

**Figure 4:**
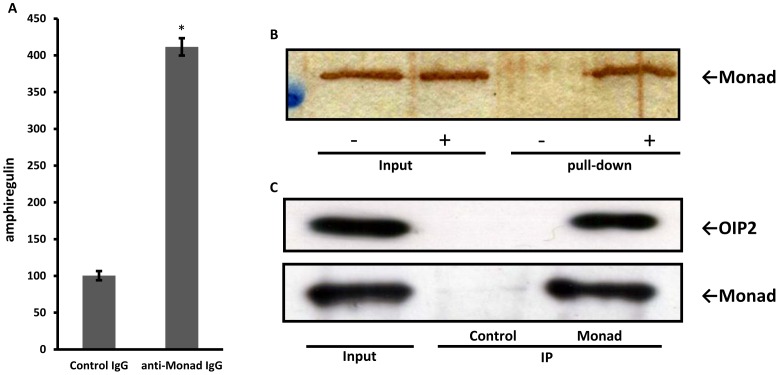
Interaction between amphiregulin mRNA and Monad. (A) The lysate from MDA-MB-231 cells were immunoprecipitated with anti-Monad antibody. Normal rabbit IgG was used as control. Quantitation of associated mRNA was performed using real-time-RT-PCR and normalized to GAPDH. (B) Binding of recombinant Monad to 3′-UTR of amphiregulin. After RNA pull-down assay using 3′-UTR of sense (+) or antisense (-, control) strand of amphiregulin as probes, separation by SDS-PAGE and silver staining were performed. (C) Interaction of Monad with OIP2. MDA-MB-231 lysate was immunoprecipitated (IP) with control IgG or Monad antibody. Following separation by SDS-PAGE, immunoblotting was performed using anti-OIP2 antibody or Monad antibody.

Interestingly, it has been reported that 3′-UTR of amphiregulin mRNA contains four AREs [30], suggesting that the stability of the amphiregulin mRNA could be controlled by ARE-mediated mRNA decay system. Given that Monad negatively regulates amphiregulin mRNA stability, it is possible that Monad could be involved in the mRNA degradation machinery, exosome, via ARE. To test this possibility, first, we determined the interaction between Monad and amphiregulin mRNA. RNA-immunoprecipitation (RIP) assay was performed using Monad antibodies under conditions that preserved native protein-RNA complexes, followed by detection of the amphiregulin mRNA from immunoprecipitated complexes by real-time RT-PCR. We found that amphiregulin mRNA was bound to immunoprecipitated Monad protein with a 4-fold increase compared with IgG control (Fig. 4A). The bound mRNA of MMP-1 and uPA was not different in both samples (data not shown). We next examined whether Monad directly associates with RNA. We performed RNA pull-down assays using 3′-UTR of sense or antisense (control) strand of amphiregulin as probes. Recombinant Monad protein associated with amphiregulin 3′-UTR mRNA transcribed *in vitro*, indicating that Monad harbors an RNA-binding capacity (Fig. 4B).

The results described above suggest that Monad mediates the interaction between amphiregulin mRNA and mRNA decay enzymes. Therefore, we examined whether the exosome associates with Monad. We confirmed that Monad interacts with one of the exosome components, OIP2, in MDA-MB-231 cells (Fig. 4C). Notably, this interaction was insensitive to RNase treatment (data not shown), suggesting a physical, RNA-independent interaction between Monad and the exosome.

### Effect of Monad on Cell Proliferation

**Figure 5:**
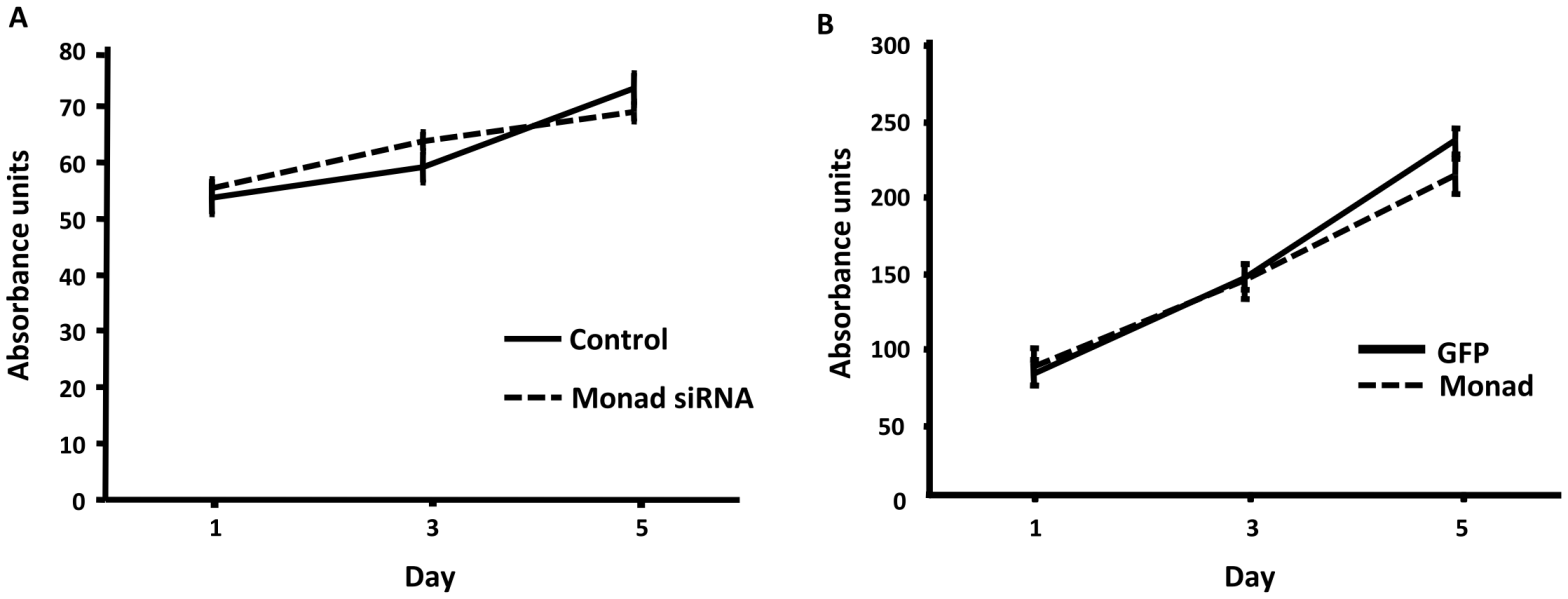
Monad does not affect the proliferation of MDA-MB-231 cells. MDA-MB-231 cells treated with control or Monad siRNA (A) or overexpressing GFP or Monad (B) were plated in 24-well cell culture plate at 2×10^4^cells per well and the proliferation was measured using the MTT assay.

We examined whether overexpression or knockdown of Monad may affect the proliferation of MDA-MB-231 cells. Minimal difference in the cell proliferation rate between control and Monad siRNA treated cells (Fig. 5A) and control and Monad-overexpressing cells (Fig. 5B) was observed, indicating that Monad does not affect the proliferation of these cells.

### Autocrine/paracrine Amphiregulin is Required for the Effect of Monad Knockdown on Invasion

**Figure 6:**
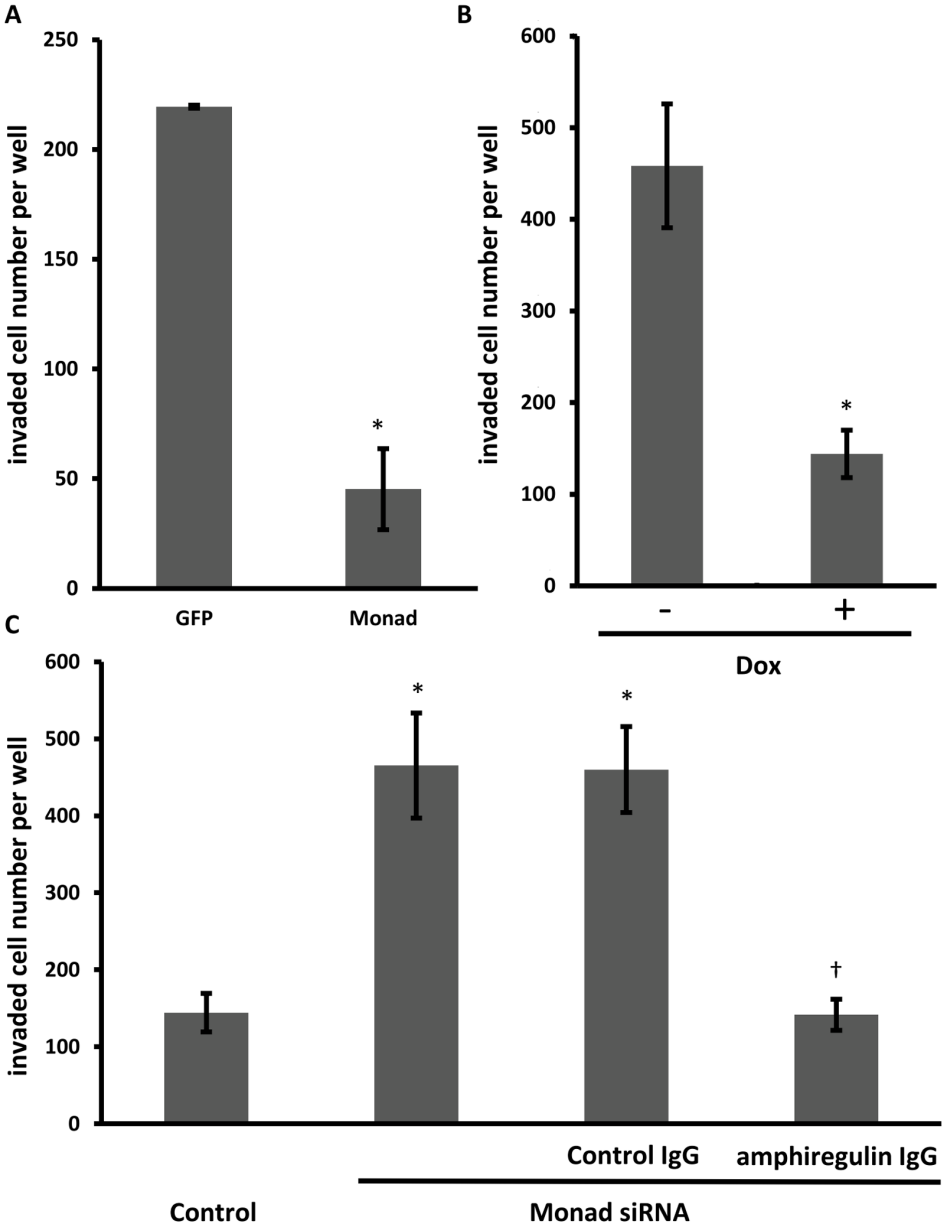
Monad regulates cell invasion of MDA-MB-231 cells. Invasiveness of GFP or Monad overxpressing MDA-MB-231 cells (A), tetracycline-regulated Monad overexpressing MDA-MB-231 cells (B), and control or Monad siRNA treated MDA-MB-231 cells (C) was assayed by a Boyden chamber method 2 days after transfection. In (B), Monad was overexpressed by doxycycline (Dox). Results are presented as means of the number of cells/well with S.E. Cells were cultured in triplicate wells/experiment and the experiment was replicated three times. (C) Knockdown of Monad increased invasiveness of MDA-MB-231 cells. Amphiregulin-neutralizing antibody (1 µg/ml) inhibited the increased invasion by Monad siRNA. *P<0.01 vs. control. ^†^ *P*<0.01 vs. control IgG.

We determined whether the overexpression of Monad may affect the invasiveness. Matrigel layer was used as a cell invasion barrier. Invasion of MDA-MB-231 cells was decreased by overexpressing Monad (Fig. 6A and B), while knockdown of Monad increased invasion of MDA-MB-231 cells (Fig. 6C). Whether increased invasion induced by Monad knockdown is specifically mediated by amphiregulin was evaluated using an antibody-blocking experiment. When the cells were treated with neutralizing antibodies against amphiregulin, increased invasion capacity of Monad-siRNA-treated cells was completely inhibited (Fig. 6C). These data suggest that autocrine/paracrine amphiregulin plays a role in increased invasion of Monad-siRNA-treated MDA-MB-231 cells.

## Discussion

Alterations in gene expression are central to the malignant phenotype. One of the molecular signatures known to be associated with poor prognosis of breast cancer is the overexpression of EGFR. Gene expression profiling studies identified the triple-negative tumor as one of the breast tumor subtypes characterized with EGFR family expression and poor prognosis [2], [3]. EGFR signaling is induced by various EGF-like growth factor family ligands such as EGF and amphiregulin. The role of amphiregulin in breast cancer cells has been suggested in previous studies. For example, it has been shown that amphiregulin increases the invasion of MCF-7 and MDA-MB231 breast cancer cells in Matrigel [31]. The EGFR family expression in breast cancer is often associated with resistance to endocrine hormone therapy [32], [33]. Amphiregulin has been found to be highly expressed by hormone therapy-resistant breast cancer cells [4]. In this study, the importance of autocrine/paracrine amphiregulin in the increased invasion of MDA-MB-231 cells by Monad knockdown was shown by Matrigel invasion assays following abrogation of autocrine amphiregulin using an antibody-blocking experiment. On the other hand, Monad was unable to affect the proliferation of MDA-MB-231 cells, which is consistent with the previous report that amphiregulin had no effect on the proliferation of MDA-MB-231 cells [31].

The exosome is a protein complex with nuclease activity that degrades RNA [18]. It contains a catalytic inactive ring-like core composed of nine subunits (Exo-9) to which two catalytic active subunits, Rrp44 and Rrp6 [34]–[37], associate. Six exosome subunits, Rrp41, Rrp42, Rrp43 (OIP2 human), Rrp45 (PM/Scl-75 human), Rrp46, and Mtr3, share sequence identity to the catalytic domains of phosphorolytic bacterial RNase PH and polynucleotide phosphorylase PNPase. Three additional exosome subunits, Csl4, Rrp4, and Rrp40, contain RNA binding domain. The exosome is present in both the nucleus and the cytoplasm [37]. The ubiquitous core of the exosome (Exo-10) is formed by Exo-9 and Rrp44. In the nucleus, the Exo-10 core recruits Rrp6 (PM/Scl-100 human).

The cytosolic function of the Exo-9 in regulating the RNA decay activities of the exosome has been a subject of interest since each of its nine subunits are essential for viability although devoid of catalytic activity. Exosomes do not bind directly to AREs but rather are recruited to ARE-containing mRNAs through interactions with ARE-binding proteins such as tristetraprolin (TTP), KSRP, and RHAU. It has been reported that TTP interacts with the human exosome component, PM/Scl-75 [38], [39] and KSRP interacts with hRrp4 [40], and RHAU interacts with hRrp40 and PM/Scl-100 [41], respectively.

In this study, we showed that Monad interacts with one of the Exo-9 components, OIP2, in MDA-MB-231 cells. Interestingly, Gonzales et al. identified yeast Pih1, also known as Nop17, as an Rrp43 (yeast homolog of human OIP2)-interacting partner by using the yeast two-hybrid system [42]. It has been shown that the deletion of Pih1 causes defects in pre-rRNA processing and box C/D snoRNA accumulation, indicating that Pih1 is required for the proper pre-rRNA and snoRNA maturation [6], [42]. Rvb1, Rvb2, and Tah1 are also involved in the process [6], [43]. This suggests that the R2TP complex and exosome could cooperatively act in various RNA metabolism pathways in eukaryotes.

**Figure 7:**
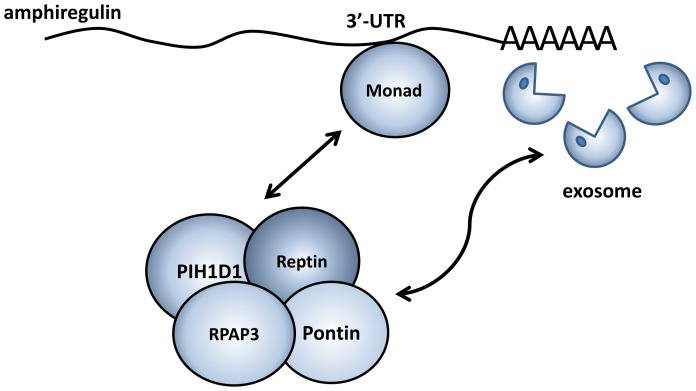
Model for R2TP complex-mediated recruitment of exosome and Monad-directed amphiregulin mRNA decay. See text for details.

Based on our new findings, we propose that Monad specifically targets amphiregulin mRNA for degradation by recruiting the exosome, and that the R2TP complex, through its chaperone-like activity, could be required for facilitating the interaction between Monad and exosome (Fig. 7).

Granneman et al. showed that WD repeat protein U3–55K interacts with the U3 snoRNA and its WD repeat domain was necessary and sufficient for the binding [44]. Similarly, our results showed that the WD repeat protein Monad directly interacts with ARE containing 3′-UTR of amphiregulin mRNA. By comprehensive approach termed “interactome capture”, Castello et al. revealed hundreds of novel RNA binding proteins, which include 23 WD repeat proteins [45]. To our knowledge, we believe that this is the first example of a WD40 repeat protein directly interacting with mRNA. Alternatively, it is also possible that Monad binds loosely defined sequences in the 3′-UTR of a large number of mRNAs, as in the case of other RNA binding proteins. The role of Monad and R2TP complex as a specific exosome recruiting factors needs to be further elucidated.

Several RNA binding proteins have been linked to the invasive phenotype in breast cancer. They have been known to be tumor-suppressive (e.g. TTP) and oncogenic (e.g. HuR), via modulation of the expression of a set of genes related to various aspects of the malignant phenotype. Monad accelerated the degradation of the pro-invasive gene, amphiregulin, as is the case with TTP, suggesting that Monad may be tumor-suppressive. Although we have previously reported the apoptosis-enhancing function of Monad [8], these results shed new light on the function of Monad as tumor-suppressor in breast cancer.

Mechanisms proposed for amphiregulin induced cellular invasion focus on the altered expression of MMPs as well as other factors involved in matrix degradation, such as uPA [46]. MMP-1, -2, -3, -8 -11, and uPA have been reported to have a highly expression in breast cancer [47]. We found that knockdown of Monad also increases MMP-1 and uPA expression. Interestingly, it has been reported that TTP also binds to the 3′-UTR in MMP1 and uPA mRNA, and accelerates the mRNA degradation in MDA-MB-231 cells [48]. This report and the present result raise the possibility that MMP-1 and uPA are also the direct targets of Monad. However, no interaction between Monad and these mRNA was observed by the RIP assay. In addition, increased invasion of MDA-MB-231 cells upon Monad knockdown was inhibited using amphiregulin antibody, suggesting that at least this effect is amphiregulin-dependent. Whether increased expression of MMP-1 and uPA is amphiregulin-dependent or not needs to be further elucidated. Notably, it has been reported that amphiregulin induced an accumulation of uPA into culture medium of MDA-MB-231 cells and that a neutralizing anti-uPA receptor antibody reversed the increased invasiveness of MDA-MB-231 cells by amphiregulin [31].

In this study, we showed that Monad-mediated degradation is one of the mechanisms that determine the stability of amphiregulin mRNA and that Monad-amphiregulin axis plays an essential role in the invasion of breast cancer cells. Considering that amphiregulin has been proposed as a biomarker for breast cancer [4], understanding Monad-amphiregulin axis in breast cancer may lead to the development of additional targeted agents to suppress this axis, and result in improved treatments for metastatic breast cancer.
